# Findings from a small-scale, exploratory content analysis of information provided to AIS patients and their parents from NHS Scoliosis Hospital Clinics

**DOI:** 10.1186/1748-7161-8-S1-O59

**Published:** 2013-06-03

**Authors:** L Arai, J Bettany-Saltikov, S Hamilton

**Affiliations:** 1 Teesside University, Middlesbrough, UK

## Background

The Patients Charter (1992) states that patients have a right to information about their condition. In patients with Adolescent Idiopathic Scoliosis (AIS) very little is known about the information provided to patients and their families. Failure to fully address patients’ and families’ health information requirements can lead to significant stress and anxiety.

## Aim

The aim of this exploratory study was to evaluate the content of hospital information material currently provided to AIS patients at the first point of diagnosis in hospital scoliosis clinics in the UK.

## Methods

Content analysis (CA) is an established and widely used method of document analysis, and can be used with quantitative or qualitative data and in a deductive or inductive way (Elo & Kyngäs, 2008). It has been defined as ‘any technique for making inferences by objectively and systematically identifying specified characteristics of messages’. The methods used here were ‘conventional’ (with a simple thematic analysis of the data) as well as ‘summative’ (though categories or key concepts were not counted but described in tabular and narrative form).

## Results

Around 31 texts from nine sources were subjected to a CA. These texts were categorised according to type, content, tone and presentation and described in tabular and narrative form. Three main observations were made about the texts: they are focused primarily on provision of medical information; that quality of life issues are peripheral within them; and that they are sometimes confused about their intended audience (and, therefore, their purpose). We do not know enough about what other information (in verbal form, written, electronic) is given to patients at the point of diagnosis. It is possible that the texts used here are provided to patients with other documents, Patients may also be accessing the Internet for help and advice. Information garnered this way may be used to complement the information leaflets described here.

## Conclusions

In many respects, the leaflets achieve their stated aim: they provide basic information to patients with AISs. The analysis here should be regarded as preliminary, and would be improved with a larger sample of (more diverse) texts from a wider range of sources.
